# A simplified mathematical model of gender-based family violence in Mexico

**DOI:** 10.3389/fpubh.2025.1565295

**Published:** 2025-05-26

**Authors:** Helena Torres Angeles, Sofia Guerrero Ruano, Laura Rocío González-Ramírez

**Affiliations:** Escuela Superior de Física y Matemáticas, Instituto Politécnico Nacional, Unidad Profesional Adolfo López Mateos, Mexico City, Mexico

**Keywords:** compartmental model, family violence, gender-based violence, public health data, violence against women

## Abstract

Gender-based violence is a major global public health challenge that persists in many societies. This study aims to assess the problem of gender-based family violence in Mexico, addressing the social neglect surrounding this issue and proposing viable strategies for its reduction. To achieve this, we analyze public health data regarding individuals who required medical attention due to injuries from family violence in Mexico, examining trends and implications. To analyze the temporal evolution of these trends we propose a simplified mathematical model to assess the problem of gender-segregated violence. This model is compartmental and consists of a system of non-linear ordinary differential equations that describe the time evolution of a gender-segregated population susceptible to violence. We employed public health data to fit the model parameters related to family violence cases and develop plausible scenarios for the evolution of the model. To evaluate the model's performance, we also analyze data related to non-family violence and self-inflicted violence. Our initial findings reveal significant differences in parameter values and the projected evolution of the population based on gender. Notably, we observe a tendency for female victims to outnumber male victims by almost two orders of magnitude in cases of family violence in Mexico. Additionally, we explore theoretical recovery measures integrated into the model to reduce the number of female victims. Our results indicate that achieving equality in the number of male and female victims of family violence is only possible if both female victims and aggressors receive rehabilitation. Therefore, this dynamic modeling approach underscores the need for public strategies that not only assist female victims in escaping violent environments, but also promote awareness and recovery programs for those who commit acts of violence.

## 1 Introduction

Gender-based violence is currently a significant global public health challenge (1, 2). Gender-based violence refers to physical or psychological aggression directed toward individuals or groups based on their sexual orientation, gender, or gender identity (3). The previous definition is ample and incorporates various forms of physical and emotional abuse. Gender-based violence impacts the identity and wellbeing of affected individuals, regardless of their gender. In this context, gender refers to the social attributes and opportunities associated with being male or female. These attributes, opportunities, and relationships are shaped and learned within society, they are specific to a given geographical area, and can change over time. Gender significantly influences what is expected, permitted, and valued in individuals (4–6).

Violence against women refers to any act of gender-based violence that results in physical, sexual, or psychological harm to women. This includes intimidation, coercion, or arbitrary deprivation of liberty, whether the incidents occur in public or private life (7–9). According to the *Violence against women prevalence estimates (2018 reports)* published by the World Health Organization (2), nearly one in three women has lived at least one incident of physical and/or sexual violence in their lifetime. Such acts are usually perpetrated by either intimate partners or non-partners. Furthermore, the number of women and girls intentionally killed in 2022 was the highest documented in the past 20 years (10). These alarming numbers have raised significant concerns about global violence against women and highlight the urgent need for international actions to prevent and eliminate this public health problem. One major challenge in understanding gender-based violence—regardless of the victim's gender identity—is the availability of reliable data that accounts for it. Indeed, due to geographical or technical limitations in data collection, the statistical analysis of such data may be constrained. Consequently, organizations such as the UN Women and the World Health Organization (11, 12), are working to raise awareness about the importance of accurate data collection. This challenge is particularly present in developing countries, where funding limitations prevent the collection of thorough datasets that document incidents of violence (2, 10).

According to *The Council of Europe Convention on preventing and combating violence against women and domestic violence* (13) among the different forms of reported gender-based violence, we can find psychological violence, physical violence, forced marriages, sexual violence, genital mutilation, and sexual harassment, among others. These forms of violence can be categorized into five interrelated types: physical violence, verbal violence, psychological violence, sexual violence, and socioeconomic violence (6). Additionally, there are other forms of violence, such as domestic or family violence, which may involve a combination of the five types of violence previously mentioned. In this context, family violence refers to abuse perpetrated by a family member, while domestic violence pertains to abuse committed by an intimate partner. These different types of violence can occur in both private and public spheres and may be carried out by individuals or groups (such as organizations or institutions) (6). Given the complex and multifactorial components of gender-based violence, along with existing data collection limitations, accurately estimating the number of individuals affected by various forms of violence in public spaces remains a significant challenge.

In Mexico, violence against women is a neglected social and public health problem that has been reasonably documented (14–16). The origins of this problem can be traced back to the multicultural origin of the country (17). A common argument for underestimating the problem of violence against women in Mexico is the belief that men experience violence at comparable rates. To examine this argument, a rigorous meta-analysis explored the prevalence of intimate partner violence among Mexican men and women (18). The findings revealed that intimate partner violence affects both genders and is a significant public health issue, influenced by demographic and situational factors. Some women affected by violence seek medical attention in public health centers for injuries inflicted by their aggressors. In certain instances, these women approach authorities to initiate legal proceedings by filing a complaint, following the process outlined in the Organic Law on Women's Right to a Life Free of Violence, enacted in May 2008 (19). However, many women who experience gender-based violence may not seek medical services or pursue legal actions. As a result, official reports on female victims of violence significantly underestimate the actual numbers. To better understand the complexities of violence against women in Mexico, efforts have been made to identify factors that prevent victims from leaving their violent environments. Among these factors are the social values shared by victims and their communities (20, 21). The social and economic status of the victims can also play a crucial role in the occurrence of violence. According to the United Nations Children's Fund in Mexico (UNICEF Mexico), 50 percent of young people live in poverty, and of those, 63 percent have experienced some form of abuse. Furthermore, in 2022, reports indicated that family violence in Mexico had increase by 24 percent compared to 2020 (22). Consequently, it is essential to identify and differentiate the underlying factors within Mexican society that enable various forms of violence against women, men, and children to persist. Additionally, raising awareness about this pressing public health problem is essential.

Mathematical models have been used to understand various social and public health challenges. Among the different types of mathematical models, compartmental models are particularly important. These models were developed in the beginnings of the 20th century to understand the evolution of infectious diseases (23, 24). Their foundation involves dividing the studied population into distinct compartments. Under specific assumptions, the interaction and exchange of individuals between the different compartments is allowed. For example, during the COVID-19 pandemic, the *SIR* model and its variants were widely used to describe the dynamics of a susceptible (*S*), infected (*I*) and recovered (*R*) populations impacted by the SARS-CoV-2 virus. This approach proved to be highly valuable in raising awareness about necessary precautions and restrictions, such as the declaration of a health emergency by the United Nations, the implementation of quarantines, borders closures in some countries, and the mandatory use of face masks to mitigate transmission (25–29). In a more targeted context, mathematical compartmental models have also been employed to examine violence as a public health problem in various scenarios (30–34). However, a significant limitation of this modeling approach, is the lack of high-quality data, which is crucial for estimating model parameters and validating the models. As previously mentioned, various factors, can lead to inadequate data collection on violence incidents. For instance, many cases of violence go unrecorded because victims may be reluctant to seek help from hospitals due to fear, may downplay the severity of the situation, or may simply lack the means to report it. As a result, the accuracy and usefulness of mathematical models increase when data is detailed, collected frequently, and allows for effective parameter estimation and model validation. This, in turn, leads to more reliable projections and facilitates in the development of strategies to mitigate specific instances of violence. For example, in Gonsalves et al. (30) a mathematical model indicated that increasing the availability of toilets in Khayelitsha, South Africa, could help reduce sexual violence.

The aim of this work is to develop a mathematical model that addresses gender-based violence in Mexico in a simplified context. In Section 2, we analyze public data related to this issue by examining the number of hospital admissions for various forms of violence, categorized by gender. Specifically, we focus on family violence, non-family violence, and self-inflicted violence. We justify that family violence is a form of gender-based violence, while non-family violence and self-inflicted violence are not included in this classification. In Section 3, we propose a simplified compartmental model of gender-segregated violence. Our model is general and can be used to specifically describe gender-based violence. The compartments in this model represent a population of both susceptible individuals and aggressors (without distinguishing gender), as well as a gender-segregated population that experiences violence. We propose various scenarios using data about the Mexican population and estimate the model parameters based on public health statistics. We specifically focus on gender-based family violence in Mexico. We have developed this model based on trends found in public health data as this is the most tangible way to provide scientific evidence that family violence in Mexico remains a critical issue and further actions need to develop strategies aimed at mitigating this problem. Our analysis considers data from 2010 to 2022, and we use data from 2023 to evaluate the performance of our gender-segregated violence model. We discuss the implications of the model parameters and examine long-term projections under these configurations. Additionally, we propose different theoretical recovery mechanism for individuals affected by violence as well as for aggressors and analyze the dynamics of the model under these mechanisms. The proposed recovery mechanism can translate into public strategies aimed at combating gender-based family violence in the country. Finally, in Section 4 and Section 5, we discuss the implications, limitations, and opportunities for improvement of the model, along with our conclusions. In the Appendix, we explore the qualitative features of the model and establish possible dynamics based on different parameter configurations.

## 2 Materials and methods

### 2.1 Public health data on gender-segregated violence in Mexico

**Figure 1:**
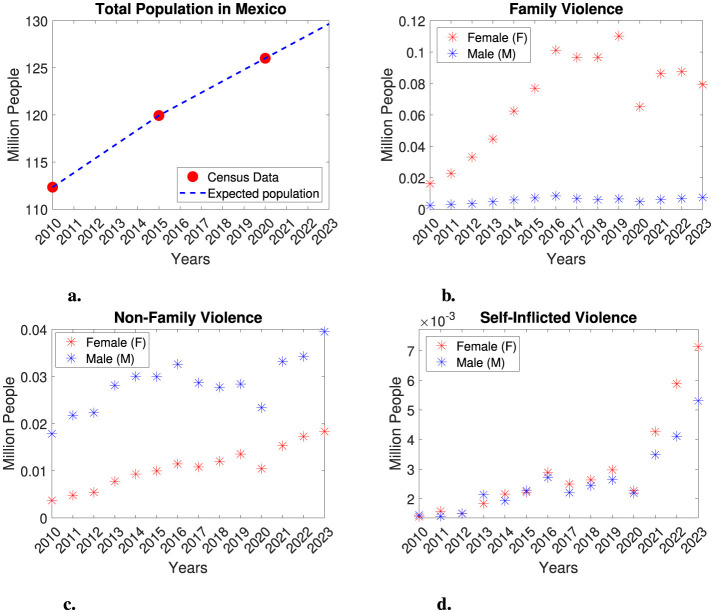
Total population in Mexico and individuals seeking medical attention due to violence. **(a)** Total population in Mexico for the years 2010, 2015, and 2020, along with interpolation data. **(b–d)** Individuals seeking medical attention in Mexico due to violence. The number of women and men (in millions) is represented by red and blue asterisks, respectively. **(b)** Family violence. **(c)** Non-family violence. **(d)** Self-inflicted violence.

To better understand gender-based violence in Mexico, we consulted databases from the National Institute of Statistics and Geography (INEGI—Instituto Nacional de Estadística y Geografía, in Spanish). INEGI is an autonomous government agency that is responsible for coordinating the collection of statistical and geographic information about Mexico. The Integrated System of Statistics on Violence against Women (SIESVIM - Sistema Integrado de Estadísticas sobre Violencia contra las Mujeres, in Spanish), is an online platform developed by INEGI that provides public access to violence indicators in Mexico. Before selecting a database, we conducted an in-depth analysis of the various violence indicators available in SIESVIM. We found that some databases lacked complete temporal information. For instance, some violence indicators were measured only every 4 years, and certain indicators did not include gender information, even though a high percentage were assumed to represent females. As a result, we selected a database that was complete, contained gender-segregated information, and included data measured annually over more than a decade. The selected dataset is titled *People Treated in Medical Units, According to the Intentionality of the Injury* and it is found under the section *Institutional Actions* and the subsection *Violence Care Services*. This dataset provides gender-segregated information on the number of individuals treated in medical facilities, categorized by the intentionality of their injuries, which includes family violence, non-family violence, and self-inflicted violence. Data for this dataset were collected annually from public and private medical institutions across Mexico from 2010 to 2023. The victims included in the dataset were admitted to various healthcare facilities, such as the Mexican Social Security Institute (IMSS—Instituto Mexicano del Seguro Social), the Institute of Social Security and Services for State Workers (ISSSTE- Instituto de Seguridad y Servicios Sociales de los Trabajadores del Estado), the Ministry of National Defense (SEDENA—Secretaría de la Defensa Nacional), Mexican Petroleum (PEMEX—Petróleos Mexicanos), private insurance companies, the Ministry of the Navy (SEMAR—Secretaría de Marina), Federal Government Health Programs, and the Popular Insurance/Health Institute for Wellbeing (INSABI- Instituto de Salud para el Bienestar). For simplicity, we will refer to this dataset as *Individuals Who Required Medical Attention Due to Violence* throughout the remainder of this manuscript. In addition, we will also use reported estimates of the total population of Mexico from 2010 to 2020 (35). Please refer to Table 1 and Figure 1 for further details.

**Table 1:** Individuals in Mexico who required medical attention due to various types of violence, including family violence, non-family violence, and self-inflicted violence (38).

	**Family violence**		**Non-family violence**		**Self-inflicted**	
**Year**	**Women**	**Men**	**Women**	**Men**	**Women**	**Men**
2010	16,240	2,395	3,703	17,850	1,395	1,449
2011	22,765	2,983	4,790	21,775	1,582	1,410
2012	33,177	3,491	5,428	22,299	1,505	1,501
2013	44,683	4,686	7,763	28,114	1,837	2,134
2014	62,434	5,902	9,302	30,034	2,158	1,930
2015	76,934	7,139	9,976	29,963	2,224	2,271
2016	101,178	8,458	11,453	32,612	2,886	2,721
2017	96,651	6,695	10,818	28,654	2,497	2,210
2018	96,700	6,018	12,038	27,731	2,635	2,440
2019	110,210	6,538	13,541	28,410	2,974	2,642
2020	65,242	4,702	10,432	23,403	2,271	2,191
2021	86,284	6,040	15,286	33,190	4,264	3,487
2022	87,591	6,782	17,275	34,268	5,879	4,113
2023	79,295	7,485	18,354	39,497	7,132	5,305

The dataset, titled *Individuals Who Required Medical Attention Due to Violence*, allows us to measure the number of women and men who required medical assistance as a result of violent events, along with their sociodemographic characteristics. It provides statistical information on aggressors, incidents, injuries, and the consequences of violence, as well as the healthcare services provided. This dataset has been systematically analyzed, during which some errors were identified in the cross-referencing of variables such as “date of occurrence” and “date of attention”. Additionally, unspecified values related to aggressors are present in the data, and cases involving multiple aggressors are not clearly defined. In Table 1, we present gender-segregated data according to injury types: family violence, non-family violence, and self-inflicted violence. The original dataset also included information on accidental injuries, which we have excluded from this study. A visual examination of Table 1 reveals a notable difference in the prevalence of family and non-family violence injuries between genders, while no significant differences are observed for self-inflicted injuries. It is crucial to recognize that family violence incidents constitute a form of gender-based violence, as data indicate a higher prevalence among women. Conversely, non-family violence incidents are associated with various factors and can involve aggression by any individual or organization (e.g., public altercations, workplace violence, involvement with drug cartels, etc.). While non-family violence does not inherently imply gender-based violence, it still reflects a form of gender-segregated violence. Notably, in the case of family violence injuries, the data shows a significant difference in the number of women affected compared to men, with a one to two order of magnitude difference. Therefore, within this representative dataset, we find that women in Mexico are more likely to sustain injuries due to family violence than men. As a result, we are particularly interested in examining this evidence of gender-based family violence. It is also essential to note that the dataset refers to cases of “extreme violence”, where the injuries of the victims were severe enough to require medical assistance. Unfortunately, in many cases of gender-based violence, victims do not seek healthcare services or report incidents to authorities, making it difficult to determine the actual number of victims. Consequently, we might expect that the gender-segregated number of victims of family violence is significantly higher. For non-family violence, the number of female and male victims is comparable, with men sometimes showing a one order of magnitude difference compared to women. This indicates a higher prevalence of non-family violence against men; however, women are also victims of this type of violence. In the case of self-inflicted violence, no clear differences between genders are observed.

In Figure 1, we present a schematic representation of the information found in Table 1 along with estimates of the total population in Mexico. For our modeling purposes, we express the number of victims in millions. In Figure 1b, we display data on female and male victims of family violence. For male victims, we observe a relatively stable trend, with numbers consistently remaining below 0.01 million from 2010 to 2023. In contrast, the number of female victims exhibits an overall increasing trend from 2010 to 2019. However, there is a sudden decrease in 2020, which reduces the number of victims down to levels similar to those of 2014. From 2021 to 2023, there is a slight increase in female victims again. Notably, the decrease in victims observed in 2020 coincides with the COVID-19 pandemic. We hypothesize that the technical difficulties arising from the pandemics—such as quarantines, containment measures, and the discontinuation of public programs—may have contributed to the decline in the reported number of female victims, leading to potential underreporting during this period. However, in the modeling framework we will develop in this study, we will also examine the possibility that this decrease represents a recovery trend for female victims of family violence. In the case of non-family violence, we observe an overall increasing trend for both female and male victims, with males experiencing a higher prevalence of violence. However, the difference between the two groups is less significant compared to family violence cases. We also note a significant decrease in the number of non-family violence victims in 2020, which parallels the decline in female victims of family violence. We attribute this trend to the impact of the COVID-19 pandemic on health services and data collection. Following this decrease, there is a rise in the number of victims. Lastly, we observe a complicated trend regarding self-inflicted violence for both groups. Initially, there is a slow increase in the number of victims, followed by a decrease in 2020—again likely due to the pandemic—before experiencing a rapid rise in the number of victims. For further details, please refer to Figure 1.

### 2.2 A simplified mathematical model of gender-segregated violence

We propose a simplified mathematical model to further analyze the data presented in the previous section. Our goal is to develop this model based on reasonable, although limited, assumptions. In García-Hernández (36), a simplified version of this model was used, without gender segregation, to describe the number of individuals who required medical assistance through Mexican emergency telephone lines. In this study, we will establish a gender-segregated violence model. Our work aims to replicate the previously presented data on *Individuals Who Required Medical Attention Due to Violence*, examine whether the model can identify differences in violence directed toward women and men, and project the evolution of various types of violence. We emphasize our particular interest in understanding the significant disparity in family violence against Mexican women, as we seek to provide additional evidence to combat the denial of this social problem. The model is based on the following assumptions:

- The total population is divided into compartments: aggressors, susceptible individuals, non-susceptible individuals, male violence victims, and female violence victims. It is assumed that non-susceptible individuals do not influence the evolution of violent situations.
- Both the aggressor and susceptible compartments include individuals of both genders.
- The aggressor compartment evolves according to a saturated growth dynamics due to the limitation of resources (i.e., a logistic growth model). As a result, we do not consider the explicit birth or death of new individuals.
- The susceptible compartment also evolves under saturated growth. However, when aggressors and susceptible individuals interact, some susceptible individuals may become victims of violence. This transition moves susceptible individuals into new compartments for male or female victims of violence. We assume that these interactions have a negative proportional effect on the rate of change of susceptible individuals. These interactions are modeled using the mass action law, which states that the interaction rate is proportional to the number of aggressors and susceptible individuals present.
- The compartment for male and female victims of violence evolve from the transition of susceptible individuals into individuals who are victims of violence. In these compartments, we assume that individuals may cease to exist due to violence or natural causes.

**Figure 2:**
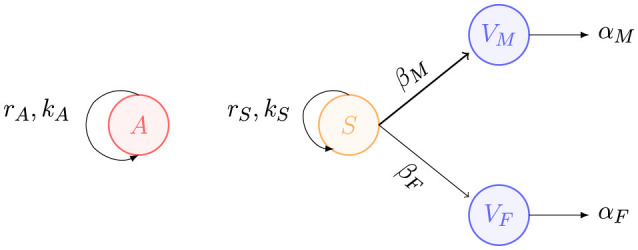
A simplified compartmental model for gender-segregated violence.

We mathematically express the previous assumptions and establish our gender-segregated violence model using the following compartmental system of ordinary differential equations:


(1) $$\begin{array}{l} \;\frac{dA}{dt}=r_{A}A(1-\frac{A}{k_{A}})\\ \;\frac{dS}{dt}=r_{S}S(1-\frac{S}{k_{S}})-\beta_{M}AS-\beta_{F}AS\\ \frac{dV_{M}}{dt}=\beta_{M}AS-\alpha_{M}V_{M}\\ \frac{dV_{F}}{dt}=\beta_{F}AS-\alpha_{F}V_{F}, \end{array}$$

where *A* is the population of aggressors, *S* is the susceptible population, *V*_*M*_ represents male victims, and *V*_*F*_ represents female victims. In this model, the subscripts *M* and *F* refer to parameters specific to the male and female victim populations, respectively. Time *t* is measured in years, and populations are measured in millions of people. All model variables are functions of time, that is, *A* = *A*(*t*), *S* = *S*(*t*), *V*_*M*_ = *V*_*M*_(*t*) and *V*_*F*_ = *V*_*F*_(*t*). All model parameters are assumed to be positive numbers. The parameters *r*_*A*_ and *r*_*S*_ represent the growth rates of the aggressors and susceptible populations, respectively. The parameters *k*_*A*_ and *k*_*S*_ denote the carrying capacities of the aggressors and susceptible populations, respectively. The parameters β_*F*_ and β_*M*_ represent the interaction rates between susceptible individuals and aggressors, leading to male or female victims. These rates are defined as the probability, within a given time unit, that an aggressor will commit extreme physical violence against a susceptible per time unit. Several factors influence these rates, including: the social environment, e.g., populations living in a conservative or liberal society; geographic location, e.g., susceptible individuals living in a city or town; economic background, e.g., individuals with degrees of study vs. those without; contact rate, e.g., individuals staying at home or going to work all day; probability of aggression, which can be triggered by external factors such as economic crises, pandemics or job loss; health issues, e.g., disabilities of victims or substance abuse by aggressors; among others. In a manner like various epidemic models, we assume a homogeneous mixing assumption, which establishes that transmission occurs through direct contact between aggressors and susceptible. The parameters α_*M*_ and α_*F*_ represent the death rate of victims, due to natural causes or violence, separated by gender. It is important to note that the differential equation describing the dynamics of the aggressors is uncoupled from the rest of the compartments. This proposal simplifies the complexity of reality, as the behavior of the aggressor population could exhibit more complex dynamics. In this simplified model, we do not differentiate between the various types of violence. Furthermore, we consider that both the aggressors and the susceptible population consist of individuals of both genders. Please, see Figure 2, for a schematic description of the components of the model.

### 2.3 Parameter estimation and model validation

We now proceed to estimate the model parameters based on the violence dataset concerning *Individuals Who Required Medical Attention Due to Violence*. Specifically, we focus on the gender-segregated data previously presented from 2010 to 2022 for this parameter estimation. The data from 2023 was used to assess the performance of the model. The numerical procedure was carried out for each type of violence, including family violence, non-family violence, and self-inflicted violence. Data for aggressors and susceptible populations were analyzed under various scenarios, considering different percentages of the total population in Mexico. For example, the first scenario assumed that aggressors represented 20% of the total population, while susceptible individuals constituted 40%. The second scenario considered aggressors as 40% and susceptible individuals as 20% of the total population. The third scenario proposed that aggressors represented 5%, while susceptible individuals represented 30% of the total population. In all three scenarios, consistent qualitative results were obtained, and further analyses considering varying percentages yielded similar results. The initial optimized parameters included *r*_*A*_ and *k*_*A*_. These were obtained by minimizing the least squares error function of the explicit solution for aggressors, determined by equation (1) in the Appendix, while varying the percentage of the total Mexican population from year 2010 to 2022 across different scenarios. The remaining parameters (*r*_*S*_, *k*_*S*_, β_*M*_, β_*F*_, α_*M*_ and α_*F*_,) were obtained through a numerical routine developed specifically for our model in MATLAB (37). To improve the fit of the model to the data, the initial conditions for each population were also optimized. Additionally, the death rates for both male and female victims was bounded to avoid results with extreme number of deaths. The developed routine consisted of numerically solving the ordinary differential equation system and employing the predetermined lsqcurvefit function to minimize the non-linear least-squares difference between the model and the dataset. The primary objective of this parameter estimation was to replicate the dataset and identify the characteristics of the model for each type of violence. In particular, we sought to determine whether the model could detect differences in the gender-segregated interaction rates across various types of violence and project different dynamics based on gender. It is important to note that the model may have difficulties to accurately describe self-inflicted violence, as this case involves both the aggressor and the susceptible population coinciding, which does not align with the model's assumptions. However, we anticipate that the model can adequately describe cases classified as family violence and non-family violence. The model's validation was conducted by running the model until the year 2023 using the estimated parameters from 2010 to 2022. Additionally, we developed numerical routines to estimate parameters using data up to 2023, which also yielded consistent qualitative results (not shown here).

## 3 Results

### 3.1 Model performance for gender-based family violence in Mexico

**Figure 3:**
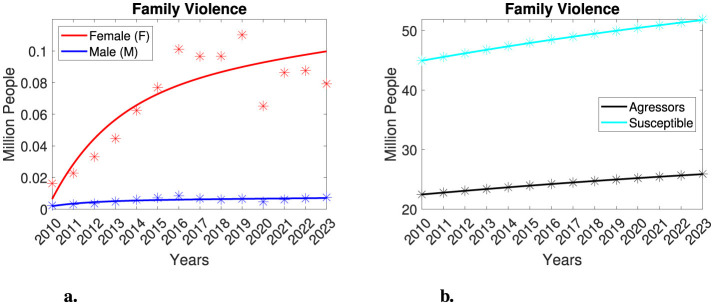
Gender-based family violence in Mexico, considering 20% of the total population as aggressors and 40% as susceptible. The asterisks represent the dataset, and the lines represent the model. Female victims are indicated in red color, while male victims are shown in blue. Aggressors are depicted in black, and susceptible individuals are represented in cyan. **(a)** Gender-based family violence model. **(b)** Aggressors and susceptible individuals.

To analyze family violence in Mexico, we estimated model parameters across several scenarios, as previously mentioned. Here, we discuss the quantitative results obtained from the first scenario. Figure 3 illustrates the performance of the model. The model adequately approximated the populations of both female and male victims and described the aggressors and susceptible populations nearly perfectly from 2010 to 2023. We note that between 2020 and 2023, the model tended to overestimate the reported number of female victims. However, we will later discuss the potential for underreporting during that time period and we will examine the possibility that this trend may indicate that female victims are recovering from family violence. In Table 2, we present the estimated parameters for this first scenario. Under this parameter configuration the victim populations are expected to increase and eventually stabilize at 9, 988 male victims and 145, 262 female victims (see Appendix for details). That is, the proportion of female victims will likely be nearly two orders of magnitude greater than that of male victims. Considering different scenarios, the parameters β_*M*_ and β_*F*_ consistently differ by at least an order of magnitude, indicating that β_*M*_ <β_*F*_. This finding suggests that the interaction between susceptible individuals and aggressors results in significantly more female victims than male victims.

**Table 2:** Estimated parameters for gender-based family violence, assuming that aggressors represent 20% and susceptible individuals 40% of the total population, respectively.

**Parameter**	**Value**
*r*_*A*_	0.054631
*k*_*A*_	30.325876
*r*_*S*_	0.054604
*k*_*S*_	61.712497
β_*F*_	0.000028
β_*M*_	0.000003
α_*F*_	0.350354
α_*M*_	0.561616

### 3.2 Model performance for gender-segregated non-family violence in Mexico

**Figure 4:**
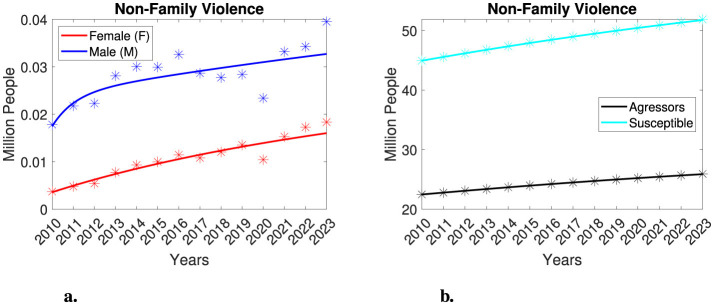
Analysis of gender-segregated non-family violence in Mexico, considering 20% of the total population as aggressors and 40% of the total population as susceptible. The asterisks represent the dataset, while the lines represent the model outcomes. Female victims are indicated in red, male victims in blue, aggressors in black, and susceptible individuals in cyan. **(a)** Gender-segregated non-family violence. **(b)** Aggressors and susceptible individuals.

We now analyze the model's performance concerning non-family violence. Similarly to our approach in studying family violence, we estimate model parameters across several scenarios, yielding consistent qualitative results. Here, we present findings from only the first scenario. Figure 4 illustrates the model's performance. The model accurately approximates each victim's population and adequately describes both the aggressors and the susceptible individuals. However, there is an overestimation of victim populations for the year 2020, followed by underestimations from 2021 to 2023 for both categories of victims. Table 3 presents the estimated parameters for this scenario. Given appropriate initial conditions, the victim populations will tend to stabilize at 45, 768 male individuals and 29, 893 female individuals, respectively. This indicates that the number of male victims is expected to be, at most, an order of magnitude greater than that of female victims. We also observe that the parameters β_*M*_ and β_*F*_ consistently differ by at least an order of magnitude, that is β_*F*_ <β_*M*_. This finding indicates that the interaction rate of the male victim's population is higher than that of the female victim's population. Since the dynamics of non-family violence do not differ dramatically between these two groups, and considering the multifactorial nature of this type of violence, we will not conduct further analysis on this case.

**Table 3:** Estimated parameters for gender-segregated non-family violence, assuming that aggressors represent 20% and susceptible individuals represent 40% of the total population.

**Parameter**	**Value**
*r*_*A*_	0.054622
*k*_*A*_	30.327267
*r*_*S*_	0.054602
*k*_*S*_	61.510064
β_*F*_	0.000002
β_*M*_	0.000023
α_*F*_	0.111478
α_*M*_	0.927221

### 3.3 Model performance for gender-segregated self-inflicted violence in Mexico

**Figure 5:**
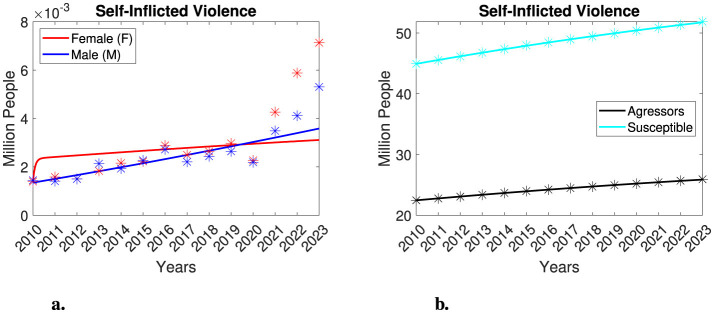
Self-inflicted violence considering 20% of the total population as aggressors and 40% of the total population as susceptible. Asterisks represent the data, while the lines illustrate the model. Female victims are shown in red, male victims in blue, aggressors in black, and susceptible individuals in cyan. **(a)** Self-inflicted violence. **(b)** Aggressors and susceptible individuals.

We implemented the model routines on the self-inflicted violence data, considering different scenarios, similar to our previous analyses. However, this time, our model was unable to accurately represent the data. As illustrated in Figure 5, the complex behavior of the data from 2010 to 2023 was not adequately captured by the model, which resulted in several underestimations and overestimation of the data during this time period. This limitation is understandable, as the model was designed within a compartmental framework where aggressors and susceptible individuals are not in the same compartment. Consequently, the inability to replicate this data can actually serve as a model validation as the data does not align with the model's assumptions. Given the inadequate performance of the model and the inconsistency of the datasets with the model's assumptions, we have decided to exclude this case from further analysis.

### 3.4 Modeling consequences of gender-based family violence in Mexico: future projections and recovery mechanisms

One significant advantage of mathematical models is their ability to analyze potential future projections developed *in silico*. This approach allows for the exploration of the dynamics of the model compartments while integrating external factors to evaluate plausible changes in the system. Therefore, in this section, we will analyze model projections and modifications, specifically focusing on the problem of family violence problem, which we consider a clear manifestation of gender-based violence. We will not be examining other types of violence, such as non-family violence and self-inflicted violence, as they require distinct analyses related to social, psychological, and geographical factors that contribute to those forms of violence. For family violence, we will also introduce theoretical recovery mechanisms to numerically assess the feasibility of equaling the number of male and female victims.

#### 3.4.1 Projections of gender-based family violence in Mexico

**Figure 6:**
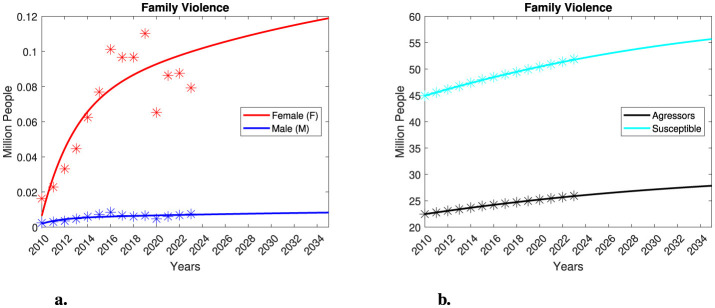
Model projection up to 2034 based on the previous parameter estimation of individuals who have experienced family violence, considering 20% of the total population as aggressors and 40% of the total population as susceptible individuals. **(a)** Gender-based family violence. **(b)** Aggressors and susceptible individuals.

In Figure 6, we present the projection of the family violence model up to the year 2034, based on previously estimated parameters. The analysis indicates that both the male and female victim populations will continue to increase; however, the increase rate for male victims will be significantly slower compared to that of female victims. Notably, for the chosen parameters, the model forecasts a continuous increase in family violence victims until reaching saturation points of 9, 988 for male victims and 145, 262 for female victims. Additionally, the model underscores a substantial gender-based disparity in the progression of this public health issue, with female victim population tending to be nearly two orders of magnitude larger than that of males.

#### 3.4.2 A gender-based family violence model including a recovery mechanism for female victims

**Figure 7:**
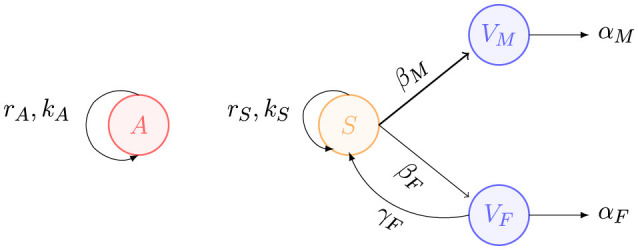
A simplified compartmental model for gender-based family violence, including a recovery term for female population.

The previous section revealed important features of the evolution of gender-based family violence in Mexico. To enhance our proposed model, we now focus on the evolution of female victims while considering a recovery mechanism. This approach maintains the assumptions outlined in our simplified mathematical model of gender-segregated violence, but incorporates a theoretical recovery component. This adjustment implies that, influenced by external factors such as awareness campaigns, shelters for battered women, and referrals to public instances, female victims can transition back to the susceptible compartment. We introduce a parameter γ_*F*_, representing the recovery rate of female victims, assuming that the recovered population is proportional to itself by a factor of γ_*F*_. Additionally, we will also explore whether the reported decrease in female victims in 2020 reflects actual recovery rather than being solely a consequence of COVID-19 pandemics, as previously justified. The female recovery model is expressed as follows:


(2) $$\begin{array}{l} \;\frac{dA}{dt}=r_{A}A(1-\frac{A}{k_{A}})\\ \;\frac{dS}{dt}=r_{S}S(1-\frac{S}{k_{S}})-\beta_{M}AS-\beta_{F}AS+\gamma_{F}V_{F}\\ \frac{dV_{M}}{dt}=\beta_{M}AS-\alpha_{M}V_{M}\\ \frac{dV_{F}}{dt}=\beta_{F}AS-\alpha_{F}V_{F}-\gamma_{F}V_{F}, \end{array}$$

where the variables and parameters align with those in the gender-segregated violence model, with the addition of a recovery rate determined by γ_*F*_. The compartments and relationships depicted in this model are illustrated in Figure 7.

We now assume that the dynamics governing our gender-segregated violence model determine the evolution of compartments from 2010 until a designated *recovery year*, which can be set to 2020, 2021, 2022 or 2023. Starting in the *recovery year*, the recovery mechanisms are activated, and the dynamics are now determined by our female recovery model. Our objective is to analyze how the compartments evolve during this recovery process. We maintain the rest of the parameters at values determined during our previous parameter estimation while uniquely varying the parameter γ_*F*_ and the *recovery year*. We intend to investigate the feasibility of recovering a substantial number of female victims to significantly decrease the population in this compartment. Ideally, we aim to determine if there exists a recovery rate that leads to an equal number of female and male victims.

**Figure 8:**
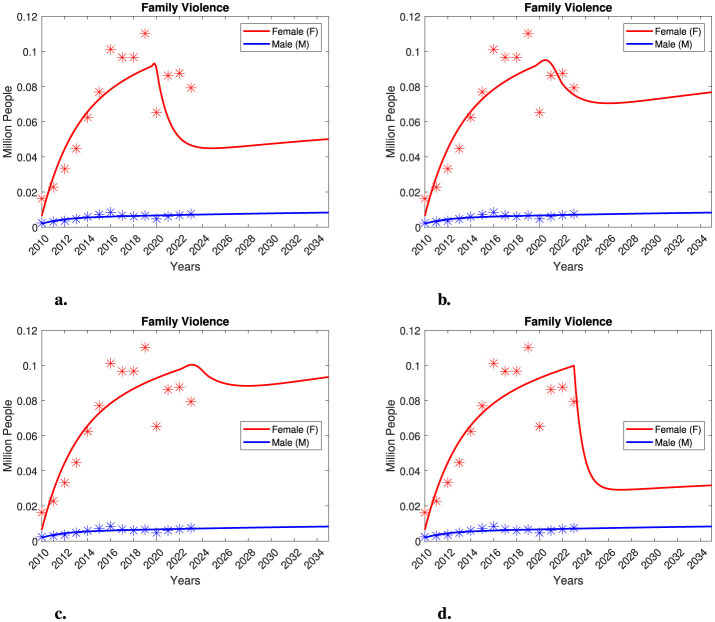
Recovery model projections considering 20% of the total population as aggressors and 40% of the total population as susceptible, respectively. The model was simulated using various recovery parameter values for γ_*F*_, and different *recovery years* as follows: **(a)** γ_*F*_ = 0.5 with the *recovery year* set to 2020, **(b)** γ_*F*_ = 0.2 with the *recovery year* set to 2020, **(c)** γ_*F*_ = 0.1 with the *recovery year* set to 2023, and **(d)** γ_*F*_ = 1 with the *recovery year* set to 2023.

In Figure 8, we present our findings from the female recovery model. We obtained similar qualitative results by varying both the *recovery year* and the recovery rate, γ_*F*_. In Figure 8a, we assume that the decline in the number of female victims observed in 2020 was due to the initiation of a recovery process commencing that year. When the female recovery rate exceeds the female death rate, we observe a sudden decrease in the number of victims. However, these dynamics represent a significant underestimation of the data reported from 2021 to 2023. Even with this recovery process, it is unlikely that female and male victim numbers will equalize within a decade. In Figure 8b, we initiated the recovery process in 2021 with a recovery rate similar to the female death rate. This approach provides a strong fit for the reported victim numbers from 2021 to 2023. Nonetheless, following an initial decline in female victims, we see an eventual increase, albeit at a slower rate. Thus, even in this case, we again observe a trend indicating higher prevalence of family violence among females. In Figures 8c, d, we initiated the recovery process in 2023, employing recovery rates differing by an order of magnitude. In Figure 8c, the recovery rate is on the same order of magnitude as the female death rate; nevertheless, the evolution of the number of female victims is not optimistic. Lastly, in Figure 8d, the recovery rate is an order of magnitude higher than the death rate. Here, we observe a significant decline in female victims that aligns with the data reported for 2023. Overall, there is notable tendency toward a significant reduction in female victims. However, even under conditions of an extremely high recovery rate, cases of female victims still outnumber those of male victims. Therefore, our analysis indicates that achieving equality between female and male victims of family violence within a short time frame is highly improbable unless an exceptionally high recovery rate is achieved. In particular, we note that to equalize male and female victims, a recovery rate at least two orders of magnitude higher than the female death rate would be necessary. This finding emphasizes the importance of not neglecting this problem and highlights the urgent need to develop effective recovery strategies. In the following section, we will explore additional recovery mechanisms for our model.

#### 3.4.3 A gender-based family violence model including recovery mechanisms for female victims and aggressors

**Figure 9:**
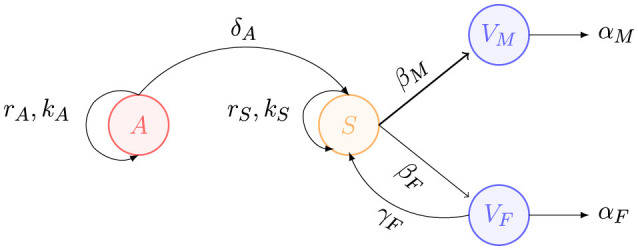
A simplified compartmental model for gender-based family violence including recovery terms for female victims and aggressors.

The previous section highlighted the challenges of recovering female victims, noting that considering various recovery rates and recovery starting times did not permit to achieve equality between male and female victims within a decade. In particular, we found that even when female victims began to recover, their progress was often short-lived unless we implemented an unrealistically high recovery rate that exceeded the death rate. Now, we aim to explore a complementary recovery mechanism focused on the aggressor population. Our objective is to analyze whether it is feasible to achieve adequate recovery for both male and female victims by directing recovery efforts toward aggressors. These recovery mechanisms aimed at aggressors can be translated into public strategies such as awareness campaigns starting from a young age, psychological therapies for abusers, and public campaigns directed toward abusers, among others. That is, we aim that aggressors return to the susceptible compartment ensuring they cease their violent behavior. The proposed complete recovery model for gender-based violence is as follows:


(3) $$\begin{array}{l} \;\frac{dA}{dt}=r_{A}A(1-\frac{A}{k_{A}})-\delta_{A}A\\ \;\frac{dS}{dt}=r_{S}S(1-\frac{S}{k_{S}})-\beta_{M}AS-\beta_{F}AS+\gamma_{F}V_{F}+\delta_{A}A\\ \frac{dV_{M}}{dt}=\beta_{M}AS-\alpha_{M}V_{M}\\ \frac{dV_{F}}{dt}=\beta_{F}AS-\alpha_{F}V_{F}-\gamma_{F}V_{F}, \end{array}$$

where the variables and parameters are the same as in the gender-segregated violence model, with the addition of an aggressor's recovery rate characterized by δ_*A*_. The compartments and relationships within the model are illustrated in Figure 9.

We now assume that the dynamics described by our gender-segregated violence model govern the evolution of compartments from 2010 until a fixed *recovery year* set to 2023. Starting in the *recovery year*, the recovery mechanisms are initiated for both the female victims and aggressors and the dynamics are now determined by our complete recovery model. We set the rest of the parameters fixed at the values determined during our previous model parameter estimation while uniquely varying the parameters γ_*F*_ and δ_*A*_. Our aim is to explore the feasibility of recovering a significant number of female victims to substantially reduce the population in this compartment. Ideally, we would like to identify if there is a recovery rate that leads to a significant reduction in the female victim population and potentially balances the number of female and male victims.

**Figure 10:**
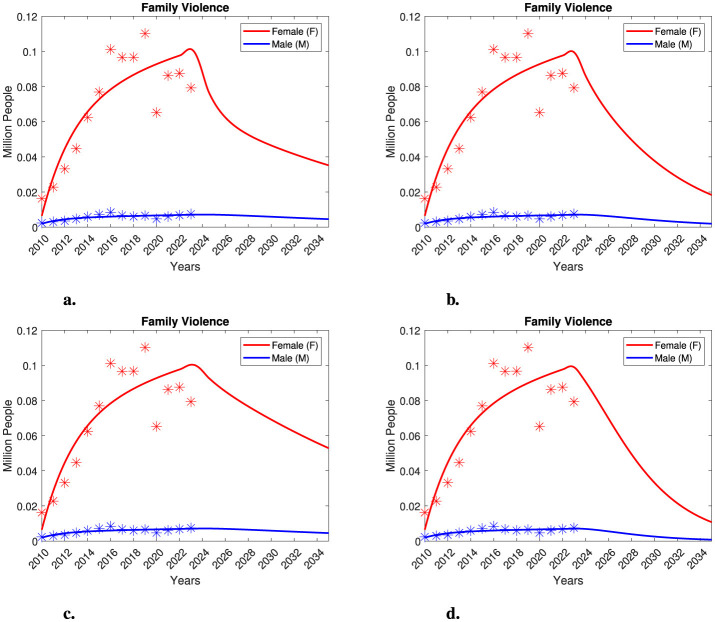
Recovery model projections considering 20% of the total population as aggressors and 40% of the total population as susceptible, respectively. The model was simulated using different values of the recovery parameters γ_*F*_ and δ_*A*_ as follows: **(a)** γ_*F*_ = 0.3 and δ_*A*_ = 0.1, **(b)** γ_*F*_ = 0.2 and δ_*A*_ = 0.2, **(c)** γ_*F*_ = 0.1 and δ_*A*_ = 0.1, and **(d)** γ_*F*_ = 0.1 and δ_*A*_ = 0.3. In all cases, the *recovery year* is set to 2023.

In Figure 10, we present our findings on the complete recovery model. We obtained similar qualitative results by varying both recovery rates γ_*F*_ and δ_*A*_. In Figure 10a, we noted that even with a low recovery rate for aggressors, there was a consistent tendency to reduce the number of female victims. In Figure 10b, we decreased the recovery rate for female victims with slightly increasing the recovery rate for aggressors, yet we still found qualitatively similar results. Figures 10c, d both considered a relatively low recovery rate for aggressors. In Figure 10c, we used a lower recovery rate for female victims compared to Figure 10d. However, in both cases, we observed a trend toward decreasing the number of female victims. Notably, in Figures 10b, d, we consistently noted a decrease in female victims alongside a significant reduction in male victims. All of the scenarios studied showed a consistent tendency to decrease the number of female victims. Therefore, our analysis indicates that achieving equality between female and male victims of family violence within a short or medium time frame is only feasible if there are sustained efforts to support female victims and provide psychological assistance or other recovery measures for the aggressors. These strategies also need to be aimed to help aggressors cease their violent behavior.

## 4 Discussion

In this work, we employ a mathematical modeling approach to analyze the problem of gender-based violence in Mexico. The presented model analyzes family violence, particularly against Mexican women, as a disease that needs to be treated accordingly to prevent significant increases in violence. Furthermore, it objectively justifies the existence of a gender-related issue in violence, helping to end the debate, usually present in Mexican society, over its credibility. By doing so, it raises awareness in society, fostering prevention efforts and eradication of this problem. We emphasize the importance of adequately collecting data to effectively use a mathematical model for projecting dynamics in public health. A clear example highlighting this point is the trend observed in the reported family violence against women in Mexico, as shown in Figure 1. The data indicates an increasing trend; however, in the year 2020, there is a noticeable sudden decrease in this trend. Nevertheless, the number of male victims does not change significantly in this year. We also observe a decline in data related to non-family violence in the same year. This marked data decline can be attributed to different factors, notably the COVID-19 pandemic, which led to the implementation of containment measures. Furthermore, the pandemic significantly impacted both public and private instances. Indeed, we analyzed the dataset in depth for specific characteristics, including year, gender, and the institution reporting the incident of violence, as well as the state in Mexico in which the incident occurred. Interestingly, as a possible consequence of the COVID-19 pandemic, there is a complete lack of reported data from state governments and private insurance for the years 2022 and 2023. These instances provided data for the years preceding 2020. Similarly, no data was reported under “non specified instances” for the years from 2021 to 2023, despite reporting data in the years prior. Several Mexican states also have no reported information from various instances from 2020 to 2023, such as IMSS and “Gratuitidad” (a federal healthcare program). Consequently, even though our gender-segregated violence model may not align precisely with the dataset from 2020 to 2023, we suspect that there has been an underestimation of the data due to all the technical difficulties stemming from the pandemic, as well as the dissolution of certain private and public programs. Thus, we argue that the model reflects a plausible trend in data related to family violence. Due to various reasons that may lead victims of family violence to refrain from reporting their cases, this study analyzes data on individuals treated in medical instances for violence-related injuries, categorized by the type of violence. It is important to emphasize that not all patients who experience family violence officially reported the abuse; some only shared the cause of their injuries with medical staff without pursuing legal action. By examining this data, we can obtain a more realistic approach, as victims are more likely to seek medical attention at a hospital than to report the incident to the authorities. Another important point to consider is that the dataset can be categorized as reflecting “extreme violence”, as physical acts of violence were significant enough to require medical attention for victims. However, this data set does not include cases of violence that do not lead to medical intervention, nor does it address other forms of violence experienced by women, such as psychological or economic abuse. Unfortunately, this model only captures these extreme cases, which means we may be underestimating the actual number of family violence victims. Nevertheless, our modeling approach can only be significant if it is restricted to tangible evidence available in public datasets. Therefore, both the dataset and the model indicate a higher incidence of violent situations against women compared to men in the case of family violence. A major consequence of this work is the necessity for ongoing efforts to maintain high standards for data collection in order to develop quality datasets. Such datasets are essential for creating accurate models that can help us understand the dynamics of public health issues and devise strategies to address the challenges posed by these problems. In particular, increasing awareness of the importance of reporting incidents of violence to public entities is vital to ensure victims receive the necessary support.

The model we developed has both advantages and limitations. Among the advantages is that we were able to adequately describe the gender-segregated violence in Mexico. This was made with a minimal model consisting of four compartments. We selected bilinear interactions among these different compartments to facilitate the mathematical analysis. This simplified model produced projections showing a tendency for higher family violence against women compared to men, while non-family appears more prevalent among men than women. These projections were based on the estimation of parameters of gender-segregated violence in Mexico. The model was unable to describe self-inflicted violence, which is reasonable, as our simplified setting does not considered a compartment of both susceptible and aggressors simultaneously. Nevertheless, a mathematical model cannot be perfect, and this model has limitations. Among the limitations is that it cannot adequately represent the dynamics specific to the year 2020, which we have justified previously. In terms of technical parameter estimation, if the death rates of the female and male populations are not bounded, then the difference between the percentages of the aggressor population and the susceptible population should not be too large, ideally it needs to remain within a range of 25–30 percentage. As a result, the model does not necessarily fit when arbitrary percentages of susceptible individuals and aggressors are chosen if there are no restrictions in the death rate. This produces unrealistic solutions with extremely large death rates. These solutions are not obtained once the death rates are bounded (to one). Furthermore, considering the aggressor population as entirely unaffected by the susceptible population is a model limitation as this population can have a very complex relationship with the susceptible individuals. It is also possible that an aggressor was previously a victim. Finally, we acknowledge that we have not considered variations in the state, age, or educational background of the victims, which are also important factors. Considering these characteristics will increase the difficulty of a mathematical model.

## 5 Conclusions

In this work, we established a simplified mathematical model to address gender-based violence in Mexico. We developed a compartmental model consisting of four populations: aggressors, susceptible individuals, female victims, and male victims. We considered that the interactions between aggressors and the susceptible population lead to the emergence of gender-segregated victims. We then utilize a dataset provided by INEGI, which includes individuals admitted to medical facilities due to injuries characterized by violence (family, non-family, and self-inflicted violence). We estimated the model parameters based on this and validated the model using data from 2023. The model effectively replicated data on family and non-family violence; however, it did not accurately represented self-inflicted cases, as these do not align with the model's assumptions that aggressors and the susceptible population are the same. Using the model parameters, we assessed family and non-family violence in Mexico. The model predicted an increase and saturation of these cases, based on the qualitative analysis of the model. Our findings reveal that in family violence cases, the interaction rate for female victims is one order of magnitude higher than that for male victims, supporting the fact that family violence against women is more prevalent than against men. Additionally, the qualitative analysis indicated that the victims population are likely to saturate, with the number of female victims being almost two orders of magnitude higher than that of male victims. In terms of non-family violence, our results demonstrated that men exhibit a one order of magnitude higher interaction rate than women, suggesting than men are more likely to be involved in non-family violence. The analysis also indicated that while male victims numbers saturate over time, the magnitude of saturation is one order of magnitude greater compared to women.

This modeling approach highlights a gender-segregated difference depending on the type of violence. To specifically address family violence against women, we proposed two different theoretical recovery mechanisms aimed at analyzing the feasibility of achieving equality between the number of male and female victims. The first strategy focuses on recovering female victims from violent situations. These recovery processes can result from the implementation of awareness campaigns, referrals to shelters for battered women, and other supportive initiatives. Our female recovery model indicated that achieving equality between male and female victims within a decade is highly unlikely unless the recovery rate is exceptionally high. Consequently, we introduced a second recovery mechanism aimed at reducing the number of aggressors. This approach can involve public strategies such as awareness campaigns, psychological therapy for abusers, and public campaigns targeting those who commit violence. In this theoretical framework, we aim for aggressors to return to a non-violent state, ceasing their abusive behavior. Our complete recovery model, which focuses on rehabilitating both female victims and aggressors, showed that even with low recovery rates, there is a tendency for the number of female victims to decrease. Therefore, our analysis indicates that achieving equality between female and male victims of family violence is feasible only if there are sustained efforts to support female victims and provide psychological assistance or other recovery measures for the aggressors. This highlights the urgent need for complete substantial reforms and targeted strategies, directed toward victims and abusers, to expedite the recovery of women affected by family violence in Mexico within a short time frame.

In conclusion, we emphasize that a mathematical modeling approach can help in developing strategies to mitigate gender-based violence. As more detailed models are created, we can plan public awareness campaigns, establish support centers for victims, and more. The development of accurate mathematical models is crucial, demanding high-quality datasets and multidisciplinary efforts that unite various fields to construct more realistic models.

## Data Availability

The original contributions presented in the study are included in the article/Supplementary material, further inquiries can be directed to the corresponding author.
