# Supplementary material for: A simplified mathematical model of gender-based family violence in Mexico
Source: Front Public Health. 2025 May 26;13:1565295. doi: 10.3389/fpubh.2025.1565295 (PMC12146359; doi:10.3389/fpubh.2025.1565295)
Supplement: Supplementary file 1 [file Presentation_1.pdf]

1 APPENDIX

1.1 Mathematical Analysis of a Simplified Gender-Segregated Violence Model

In this section, we present a qualitative analysis of our simplified gender-segregated model. This analysis provides valuable insights into the model dynamics. In the qualitative theory of ordinary differential equations, fixed-point solutions—which represent constant solutions of a system—are typically sought. In addition, we linearize around these solutions to determine the dynamics near them. This process facilitates an understanding of the relationship between parameters and distinct model solutions, enabling a meaningful analysis. Before we begin with the model analysis, we observe that the first model equation is uncoupled and can be solved directly by separation of variables. The solution establishes the number of aggressors at time t by:

$$A(t) = \frac{k_A A_0 e^{r_A t}}{k_A + A_0\left(e^{r_A t} - 1\right)}, \tag{1}$$

where A_0 is the initial number of aggressors measured at the initial time. Equation (1) represents a typical logistic growth model that is used to describe populations that saturate at a carrying capacity k_A.

We now begin the qualitative model analysis. Fixed points are obtained by setting all the model equations to zero:

$$\begin{aligned} r_A A\Big(1 - \frac{A}{k_A}\Big) &= 0, \\ r_S S\Big(1 - \frac{S}{k_S}\Big) - \beta_M AS - \beta_F AS &= 0, \\ \beta_M AS - \alpha_M V_M &= 0, \text{ and} \\ \beta_F AS - \alpha_F V_F &= 0. \end{aligned} \tag{2}$$

By simultaneously solving the previous equations, we find the following fixed points of the system:

$$\begin{aligned} P_1 &= (0,0,0,0), \\ P_2 &= (0,k_S,0,0), \\ P_3 &= (k_A,0,0,0), \text{ and} \\ P_4 &= \left(k_A, k_S\left(1 - \tfrac{k_A}{r_S}(\beta_F + \beta_M)\right), \tfrac{\beta_M k_A k_S}{\alpha_M}\left(1 - \tfrac{k_A}{r_S}(\beta_F + \beta_M)\right), \tfrac{\beta_F k_A k_S}{\alpha_F}\left(1 - \tfrac{k_A}{r_S}(\beta_F + \beta_M)\right)\right). \end{aligned} \tag{3}$$

The fixed points represent solutions that trivially satisfy the system. The point P_1 is a trivial solution where all compartments have no individuals. The point P_2 represents a solution in which all compartments, except for the susceptible population, have no individuals. In this case, the susceptible population consists of k_S individuals, which represents the carrying capacity. Similarly, the point P_3 represents a solution in which the susceptible and victims populations do not have individuals, while the number of aggressors reaches their carrying capacity. Thus, this is not a realistic solution as the only nontrivial compartment corresponds to the aggressors. Finally, the point P_4 represents a nontrivial solution in which aggressors, susceptible individuals, and victims can coexist.

The specific population sizes at this point depend on the relationship between the model parameters. Thus, the point P_4 represents the existence of victims and can be used to represent gender-segregated violence. Importantly, a relationship between the carrying capacity of the aggressors, the rate of growth of the susceptible population, and the interaction rates between the susceptible and the aggressors permits P_4 to represent positive populations. This is expressed by the condition $r_S - k_A(\beta_F + \beta_M) > 0$.

We now proceed to linearize the system around its fixed points. This proceeding is used to analyze the dynamics of a fixed point. In particular, a stable solution is obtained when all eigenvalues of the corresponding Jacobian matrix have a real part less than zero. Otherwise, if at least one eigenvalue has a positive real part, then the solution is unstable. Intuitively, in terms of the model, a stable fixed point represents that a small perturbation of the system around the solution will decay as time increases. Otherwise, perturbations will increase as time increases, and we will tend to move away from the fixed point solution. Importantly, the information about the stability/instability of a fixed point will help us determine the long-time dynamics of the model. To compute the linearization associated to the model, we define the following functions:

$$
\begin{aligned}
f_1(A, S, V_M, V_F) &:= r_A A\Big(1 - \frac{A}{k_A}\Big), \\
f_2(A, S, V_M, V_F) &:= r_S S\Big(1 - \frac{S}{k_S}\Big) - \beta_M AS - \beta_F AS, \\
f_3(A, S, V_M, V_F) &:= \beta_M AS - \alpha_M V_M, \text{ and} \\
f_4(A, S, V_M, V_F) &:= \beta_F AS - \alpha_F V_F.
\end{aligned} \tag{4}
$$

By computing the first-order approximation of the nonlinear system, we obtain the Jacobian matrix:

$$
J := \begin{pmatrix}
\frac{\partial f_1}{\partial A} & \frac{\partial f_1}{\partial S} & \frac{\partial f_1}{\partial V_M} & \frac{\partial f_1}{\partial V_F} \\
\frac{\partial f_2}{\partial A} & \frac{\partial f_2}{\partial S} & \frac{\partial f_2}{\partial V_M} & \frac{\partial f_2}{\partial V_F} \\
\frac{\partial f_3}{\partial A} & \frac{\partial f_3}{\partial S} & \frac{\partial f_3}{\partial V_M} & \frac{\partial f_3}{\partial V_F} \\
\frac{\partial f_4}{\partial A} & \frac{\partial f_4}{\partial S} & \frac{\partial f_4}{\partial V_M} & \frac{\partial f_4}{\partial V_F}
\end{pmatrix}
=
\begin{pmatrix}
r_A - \frac{2r_A}{k_A}A & 0 & 0 & 0 \\
-\beta_M S - \beta_F S & r_S - \frac{2r_S}{k_S}S - A(\beta_M + \beta_F) & 0 & 0 \\
\beta_M S & \beta_M A & -\alpha_M & 0 \\
\beta_F S & \beta_F A & 0 & -\alpha_F
\end{pmatrix}. \tag{5}
$$

We now substitute each fixed point in the Jacobian matrix to determine the stability/instability of each solution. We note that the previous matrix is a lower triangular matrix. Hence, for each fixed point, its corresponding eigenvalues coincide with the elements along the main diagonal. We first consider $P_1 = (0, 0, 0, 0)$. In this case, we obtain the following signed eigenvalues:

$$
\begin{aligned}
\lambda_1 &= r_A > 0, \\
\lambda_2 &= r_S > 0, \\
\lambda_3 &= -\alpha_M < 0, \text{ and} \\
\lambda_4 &= -\alpha_F < 0.
\end{aligned} \tag{6}
$$

Since two of the previous eigenvalues are always positive, the point P_1 is nonlinearly unstable. This implies that unless a trivial initial condition consisting of no aggressors and no susceptible individuals is chosen, the model cannot lead to the extinction of all of the compartments. These results are adequate with the expected description of the evolution of population dynamics.

We now evaluate the linearized dynamics around $P_2 = (0, k_S, 0, 0)$. In this case, the corresponding signed eigenvalues are:

$$\begin{aligned} \lambda_1 &= r_A > 0, \\ \lambda_2 &= -r_S < 0, \\ \lambda_3 &= -\alpha_M < 0, \text{ and} \\ \lambda_4 &= -\alpha_F < 0. \end{aligned} \tag{7}$$

Since one of the previous eigenvalues is always positive, the point P_2 is also nonlinearly unstable. This implies that, unless a trivial initial condition consisting of no aggressors and no victims is chosen, the model cannot lead to the extinction of all compartments with the exception of the susceptible population.

We proceed to develop the same analysis with the fixed point $P_3 = (k_A, 0, 0, 0)$. In this case, the eigenvalues are:

$$\begin{aligned} \lambda_1 &= -r_A < 0, \\ \lambda_2 &= r_S - k_A(\beta_F + \beta_M), \\ \lambda_3 &= -\alpha_M < 0, \text{ and} \\ \lambda_4 &= -\alpha_F < 0. \end{aligned} \tag{8}$$

We note that three of the previous eigenvalues are always negative and one of them, λ_2, can be positive or negative depending on the model parameters. In particular, $\lambda_2 > 0$ if $r_S > k_A(\beta_F + \beta_M)$. This implies that P_3 is unstable if the rate of growth of the susceptible population is greater than the carrying capacity of the aggressors multiplied by the sum of the interaction rates. This also implies that if this relation between the parameters is satisfied, then, unless a trivial initial condition is chosen, susceptible and victims populations cannot become extinct. On the other hand, if $r_S < k_A(\beta_F + \beta_M)$ then $\lambda_2 < 0$ and the solution P_3 is nonlinearly stable. This implies that if the rate of growth of the susceptible population is sufficiently low and for a convenient initial condition, then the susceptible and victim populations will tend to disappear.

We now evaluate the Jacobian matrix at the fixed point P_4. As previously mentioned, this solution represents the existence of nontrivial individuals in all of the compartments. The eigenvalues corresponding to this system are as follows:

$$\begin{aligned} \lambda_1 &= -r_A < 0, \\ \lambda_2 &= -r_S + k_A(\beta_M + \beta_F), \\ \lambda_3 &= -\alpha_M < 0, \text{ and} \\ \lambda_4 &= -\alpha_F < 0. \end{aligned} \tag{9}$$

In this case, also three of the eigenvalues are always negative and one of them, λ_2, can be positive or negative depending on the model parameters. If $r_S < k_A(\beta_M + \beta_F)$ then $\lambda_2 > 0$ and P_4 is unstable. We note that this condition is related to the stability condition of P_3. Implying that under this parameter condition and convenient initial conditions, solutions will tend towards P_3. This also implies that individuals in the susceptible and victims compartments will cease to exist. On the other hand, if $r_S > k_A(\beta_M + \beta_F)$ then $\lambda_2 < 0$ and P_4 is stable. We note that this condition was established to determine positive populations for the solution P_4. Under this parameter condition, there is coexistence of susceptible individuals, aggressors and victims.

The previous qualitative analysis indicates that trivial dynamics in the model—specifically, the extinction of individuals in all or almost all compartments—is only achieved under very specific initial conditions and very specific parameters. The overall dynamics of the model are nontrivial and under convenient initial conditions and interactions the different populations can coexist. Furthermore, we note that the relation between the rate of growth of the susceptible population, the carrying capacity of aggressors, and the interaction rates is crucial in the model dynamics.
